# Which low‐dose atropine for myopia control?

**DOI:** 10.1111/cxo.12967

**Published:** 2019-09-05

**Authors:** Safal Khanal, John R Phillips

**Affiliations:** ^1^ Myopia Laboratory, School of Optometry and Vision Science The University of Auckland Auckland New Zealand; ^2^ Department of Optometry Asia University Taichung Taiwan

**Keywords:** atropine, low‐dose, myopia

We face an epidemic of myopia as its global prevalence continues to rise at an alarming rate. Most myopia is associated with excessive elongation of the eye that stretches the retina and choroid and increases the risk of ocular pathologies, including retinal detachment and myopic maculopathy.^1^ Individuals with low degrees of myopia have an increased risk of developing these blinding conditions, but for those who progress to high myopia, the risks are enormous. For example, the odds ratio for myopic maculopathy is approximately two for myopia up to −3.00 D, but this increases to over 120 for −7.00 D of myopia.^1^

Atropine eye drops, along with orthokeratology and multifocal contact lenses, are the most effective treatments for slowing myopia progression. Nightly instillation of one drop of 1% atropine effectively halts the progressive increase in myopic refractive error and eye elongation relative to untreated eyes.^2^ However, adverse side effects including mydriasis, cycloplegia and accelerated progression on cessation (rebound), have limited the clinical use of 1% atropine. Consequently, interest has shifted to the use of much lower concentrations, which also appear to reduce myopia progression, although in a dose‐dependent manner.^3^, ^4^ For example, 0.01% atropine reportedly reduces the progression of myopic refractive error by almost 60 per cent in two years, with minimal side effects.^3^

The original justification for using 0.01% atropine for myopia control was based on findings from the Atropine for the Treatment of Childhood Myopia (ATOM) studies,^3^ which showed that 0.01% atropine reduced the rate of refractive progression. However, as highlighted in a recent commentary,^5^ there seems to be a puzzling disconnect, in which 0.01% atropine slows the refractive changes associated with myopia progression without slowing the abnormal eye enlargement. It is imperative that an effective myopia control intervention also slows the rate of eye elongation, in order to reduce the risks of myopia‐related pathologies in later life.

Despite the apparent discordance between axial and refractive outcomes, the use of 0.01% atropine is rapidly growing in popularity. According to the 2015 WHO report on myopia,^6^ 0.01% atropine is the most common strategy for managing childhood myopia in Asian countries like Singapore, where 0.01% atropine is a licensed therapeutic medicine. A recent report in the *Community Eye Health Journal*,^7^ widely accessible in developing countries, proposes 0.01% atropine as the recommended treatment for childhood myopia control, and in India, the Mumbai Group of Paediatric Ophthalmologists and Strabismologists also recommends 0.01% atropine for treating childhood myopia, with almost two‐thirds of paediatric ophthalmologists there routinely prescribing it.^8^ Moreover, a recent global survey among the members of paediatric ophthalmology societies found that nearly two‐thirds of members regularly prescribe 0.01% atropine to reduce myopia progression.^9^ Many hospital ophthalmology departments in Asia have switched to using 0.01% atropine, and there is increasing uptake of 0.01% atropine in private practices around the world, including Australia and New Zealand.

To date, evidence regarding the efficacy of 0.01% atropine in slowing eye growth comes from two clinical trials: the ATOM2 study^3^ and the Low Concentration Atropine for Myopia Progression (LAMP) study.^4^ In ATOM2, 400 myopic children aged six to 12 years received either 0.5%, 0.1% or 0.01% atropine eye drops nightly in both eyes for two years (phase 1). All treatments were then stopped abruptly for a year (phase 2 or washout phase) before initiating 0.01% atropine treatment for those who progressed by more than 0.50 D during the washout phase, for a further two years (phase 3).

**Figure 1:**
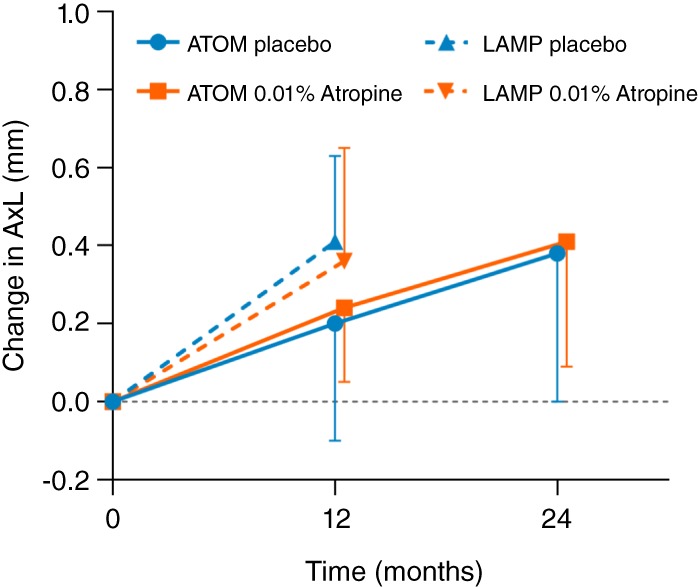
Change in axial eye length for 0.01% atropine and placebo in ATOM (0.01% data from ATOM2 and placebo data from ATOM1) and LAMP studies versus time. In both ATOM and LAMP trials, there was no significant difference between the change in axial eye length with 0.01% atropine and placebo eye drops. Error bars represent 1 SD. AxL = axial eye length.

Although 0.01% atropine was initially assumed to act as a placebo in ATOM2, it was found to produce significant clinical effects in reducing myopia progression. This serendipitous finding, which effectively resulted in the loss of the control group, forced comparisons to be made with a historical control group from a previous clinical trial (ATOM1) on 1% atropine.^3^ During the first two years (phase 1 of ATOM2), relative to the placebo group from ATOM1, 0.01% atropine significantly reduced the progressive changes in refraction (by 59.2 per cent, −0.49 versus −1.20 D), but showed no evidence of an effect on axial eye growth (data replotted in Figure 1).

Although 0.01% atropine appeared to be the most effective of the three doses in reducing refractive progression over the five‐year study duration (primarily because of the greater rebound effects with higher doses), the washout and crossover nature of the study design rendered the findings during phases 2 and 3 difficult to interpret. The use of a historical control group for phase 1 was also somewhat problematic because of differences in baseline characteristics, such as age (ATOM1: 9.20 versus ATOM2: 9.50 years), degree of myopia (ATOM1: −3.55 versus ATOM2: −4.50 D), and eye size (ATOM1: 24.8 versus ATOM2: 25.1 mm), and also axial length measurement methods (ATOM1: A‐scan ultrasound versus ATOM2: IOLMaster). However, the effect of these differences, although not quantifiable, is unlikely to have significantly influenced the findings, which indicated that over the first two years, 0.01% atropine had no effect in controlling axial eye growth.

More recently, in the double‐blind, placebo‐controlled LAMP study, 438 myopic children aged four to 12 years were randomly assigned to receive 0.05%, 0.025% or 0.01% atropine eye drops or placebo, nightly. Findings from the first year of this five‐year trial demonstrated that, compared to placebo, 0.01% atropine significantly reduced refractive progression (by 27.2 per cent, −0.59 versus −0.81 D), but had a non‐significant effect on eye elongation (0.36 versus 0.41 mm) (Figure 1). Recent two‐year results from the study suggested a maintained efficacy on refractive progression for all three concentrations.^10^ However, for ethical reasons, 0.05% atropine treatment was initiated in the control group after the first year, which ruled out the possibility of assessing the placebo‐controlled efficacy of 0.01% atropine over two years. Interestingly, 0.05% atropine eye drops significantly reduced both the rate of refractive progression (by 66.7 per cent, −0.27 versus −0.81 D) and eye elongation (by 51.2 per cent, 0.20 versus 0.41 mm) over the one‐year period and demonstrated superior efficacy to 0.025% and 0.01% at the end of two years. Despite a clear dose‐dependent effect of atropine on accommodation and pupil size, all three low doses in the trial were reportedly well tolerated, although a separate study has suggested that any concentration above 0.02% is likely to produce clinical symptoms which may pose a barrier for clinical use.^11^

Prior to the LAMP study, Huang et al.^12^ conducted a network meta‐analysis (involving direct and indirect comparisons of interventions across studies) which compared 16 different myopia interventions versus controls. Their analysis predicted a moderate treatment effect with 0.01% atropine of 0.15 mm slowing of eye elongation per year. However, the results of their study warrant cautious interpretation. By nature, network meta‐analysis provides indirect evidence, which should have concordance with available direct evidence for validity. While network meta‐analysis integrates relevant data and increases power by using pooled controls, it suffers from unknown sources of bias between pairs that are not directly compared in the original studies. At the time of the analysis by Huang et al., there were no studies directly comparing the effects of 0.01% atropine versus control. The moderate efficacy of 0.01% atropine in slowing axial elongation derived from the network analysis conflicts with the more direct evidence from both LAMP and ATOM2 studies showing no effect of 0.01% atropine, and should therefore only be regarded as indirect evidence of the efficacy of 0.01% atropine.

Despite evidence that 0.01% atropine does not slow eye elongation, it is being rapidly adopted in clinical practice and widely used – which is both unfortunate and concerning. Recent findings show that low‐dose atropine, if used in concentrations higher than 0.01%, can offer considerable slowing of myopia progression and also reduced eye growth.^4^ Both 0.025% and 0.05% atropine slow eye elongation significantly over one year (0.025%: 0.12 mm, 29.3 per cent, and 0.05%: 0.21 mm, 51.2 per cent, relative to placebo), whereas 0.01% atropine has been shown to have no significant effect in two studies (LAMP, one year: 0.05 mm, 12.2 per cent; ATOM2, two years: −0.03 mm, −7.90 per cent, relative to placebo).

**Figure 2:**
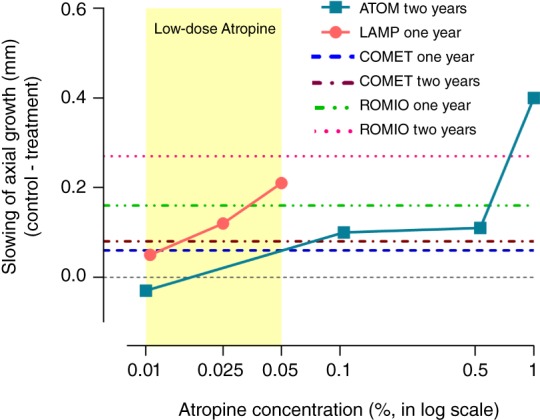
Slowing of axial growth (change of axial length in the control eye minus change of axial length in the atropine‐treated eye) as a function of atropine concentration. To facilitate comparison of myopia control therapies, the effect sizes of atropine treatments from the ATOM and LAMP studies are compared with the well‐established Correction for Myopia Evaluation Trial (COMET) data on progressive addition lens wear and the Retardation of Myopia in Orthokeratology (ROMIO) trial data on orthokeratology lens wear (plotted as dashed/dotted lines). The axial treatment effects of 0.01% atropine from both ATOM and LAMP studies are below that of the progressive addition lenses from the COMET trial, the effects of which are generally regarded as statistically significant but clinically non‐significant.

The efficacy of 0.05% atropine appears to be equivalent to that of orthokeratology lens wear for example (Retardation of Myopia in Orthokeratology [ROMIO] study, one year: 0.16 mm, 43.2 per cent; two years: 0.27 mm, 42.9 per cent).^13^ Both 0.025% and 0.05% concentrations clearly have greater efficacy than progressive addition lenses (Correction for Myopia Evaluation Trial [COMET], one year: ~0.06 mm, ~19 per cent; two years: ~0.08 mm, ~15 per cent relative to single‐vision lenses, see Figure 2), which have largely been dismissed as clinically ineffective for myopia control.^14^

Globally, over 30 registered clinical trials involving low‐dose atropine in various concentrations, ranging from 0.005% to 0.05%, are ongoing. The future results from these trials should better inform the choice of atropine concentration for myopia control and importantly, clarify the issue of rebound with discontinuation of atropine in these concentrations. Nonetheless, current evidence clearly suggests that, for pharmacological control of myopia progression, a shift in clinical practice away from using 0.01% toward prescribing 0.025% to 0.05% atropine is needed to reduce the risks of myopia‐related ocular pathologies.

In practice, initiating 0.01% atropine treatment for a child would inevitably delay implementation of an effective dose. This is particularly problematic in the early stages of myopia development when progression is most rapid. Since the sight‐threatening complications of myopia result primarily from excessive tissue stretch, effective slowing of eye growth would significantly ameliorate the risks and consequently help reduce the burden of future sight loss.
